# Investigation of the Effects of Home-Based Exercise and Cognitive Training on Cognitive and Physical Functions in Cardiac Patients: The COVEPICARDIO Study Protocol of a Randomized Clinical Trial

**DOI:** 10.3389/fcvm.2021.740834

**Published:** 2021-12-06

**Authors:** Florent Besnier, Emma Gabrielle Dupuy, Christine Gagnon, Thomas Vincent, Catherine-Alexandra Grégoire, Caroll-Ann Blanchette, Kathia Saillant, Nadia Bouabdallaoui, Josep Iglésies Grau, Béatrice Bérubé, Miloudza Olmand, Marie-France Marin, Sylvie Belleville, Martin Juneau, Paolo Vitali, Mathieu Gayda, Anil Nigam, Louis Bherer

**Affiliations:** ^1^Research Center and Centre ÉPIC, Montreal Heart Institute, Montréal, QC, Canada; ^2^Department of Medicine, Faculty of Medicine, Université de Montréal, Montréal, QC, Canada; ^3^Department of Psychologie, Université du Québec à Montréal, Montréal, QC, Canada; ^4^Research Center, Institut Universitaire de Gériatrie de Montréal, Montréal, QC, Canada; ^5^Department of Psychology, Université de Montréal, Montréal, QC, Canada; ^6^Research Center of the Montreal Mental Health University Institute, Montréal, QC, Canada; ^7^McGill University Research Centre for Studies on Aging, Montréal, QC, Canada; ^8^McGill University Department of Neurology and Neurosurgery, Faculty of Medicine, Montréal, QC, Canada

**Keywords:** cardiovascular diseases, COVID, cognition, exercise, rehabilitation, physical activity

## Abstract

**Introduction:** During the COVID-19 pandemic, confinement measures are likely to produce collateral damage to health (stress, confusion, anxiety), especially in frail individuals and those living with cardiovascular disease (CVD). In cardiac patients in particular, these measures dramatically increase the level of physical inactivity and sedentary lifestyle, which can decrease cardiorespiratory capacity and increase the risk of acute events, rehospitalization, and depressive syndromes. Maintaining a minimum level of physical activity and cognitive stimulation during the COVID-19 crisis is essential for cardiac patients. This study is designed to document the effects of 6 months of home-based physical exercise alone or combined with cognitive training on cognitive and physical functions in patients with CVD over 50 years old.

**Methods and Analysis:** 122 patients (>50 years old) with stable CVD and no contraindication to perform physical exercise training will be recruited and randomly assigned to one of the 2 following arms: (1) Home-based physical exercise alone, (2) Home-based physical exercise combined with cognitive training. The intervention lasts 6 months, with remote assessments performed prior to, mid and post-training. A follow-up 6 months after the end of the intervention (12 month) is also proposed. The primary outcome is cognition, including general functioning (Montreal Cognitive Assessment (MoCA) score), as well as performances on measures of executive functions, processing speed, and episodic memory. The secondary outcome is physical performance, including balance, gait and mobility, leg muscle strength and estimated cardiorespiratory fitness. Tertiary outcomes include mood, anxiety, and health-related quality of life as assessed by self-reported online questionnaires.

**Discussion:** With the COVID-19 crisis, there is a critical need for remote exercise and cognitive training, and to further investigate this topic, in particular for cardiac patients. The present context can be viewed as an opportunity to perform a major shift from center-based programs to home-based physical exercise. This is especially important to reach out to older adults living in remote areas, where access to such interventions is limited.

**ClinicalTrials.gov:** [https://clinicaltrials.gov/ct2/show/NCT04661189], NCT04661189.

## Introduction

Because of the COVID-19 pandemic, the population has been forced to home confinement since spring 2020, drastically reducing social interactions and altering physical activity and eating behaviors (1). This sudden lockdown radically changed the population's lifestyle (gym services are also closed), with activities mainly limited to essential needs (e.g., grocery, pharmacy, or physician visits). This confinement may also have collateral effects on cognitive and physical health (2), especially in individuals at high risk of cognitive decline like older adults (3) and patients with cardiovascular diseases (CVD) (4). Hospitals and clinics must reorganize their care services, while limiting contact between individuals. Ambulatory visits and non-essential services have been reduced, like center-based cardiac rehabilitation programs (CR) stopped or delayed, despite the fact that they are a class I level A recommendation with clinical benefits that are now well documented (5, 6). Thus, effective solutions are needed to enhance cardiovascular health and cognition in patients with CVD, while maintaining social distancing during this pandemic period. Furthermore, the pandemic context will likely persist, and full participation in center-based CR or usual exercise habits might take time or be postponed. Maintaining a minimum of physical activity during the COVID-19 crisis is essential for cardiac patients, with the advice of the medical team who could prescribe remote home-based exercise training (6, 7). Remote supervision of exercise is effective on health outcomes and safe in patients with stable CVD (6, 8–10). It is now well established that physical activity positively impacts health outcomes and cognition in older adults with CVD (11, 12). Cognitive training also enhances cognitive performances in older adults (13) and in cardiac patients post-surgery (14). Moreover, cognitive training and exercise could have a synergistic effect on cognition in healthy older adults compared to exercise training alone (15, 16). However, this synergistic effect has never been investigated in older adults with CVD. Furthermore, while there is increasing interest for multidomain interventions combining cognitive and exercise training to improve cognition in older adults, there is still a lack of knowledge on how much benefits can be gained by adding cognitive training to physical exercise, and regarding the optimal dose of each type of intervention. The COVID-19 crisis has put forward the relevance of home-based training. The present context can be viewed as an opportunity to promote a major shift in remote non-pharmacological interventional programs, so that a larger number of individuals could benefit from them.

The COVEPICARDIO project will investigate the effects of home-based physical exercise with or without cognitive training, on cognitive and physical functions in older women and men with stable CVD.

Hypotheses: 1/ After 6 months of home-based physical exercise, cardiac patients will show an improvement in cognitive (primary outcome) and physical measures (secondary outcomes), with larger gains in executive functions. 2/ Cardiac patients randomized in the combined home-based physical exercise and cognitive training group will show greater improvements in cognition and physical functions post-intervention compared to cardiac patients who did not complete the cognitive training.

## Methods and Analysis

### Study Design

The COVEPICARDIO study will be a prospective, single-blind, randomized trial with two parallel intervention arms (1:1): 1/ Physical exercise: remote monitoring and coaching of home-based physical exercise, 2/ Physical exercise, and cognitive training: remote monitoring and coaching of home-based physical exercise and cognitive training. The intervention will last 6 months. Three testing periods lasting 7 to 10 days each will be performed remotely at baseline, three (mid-training) and 6 months (post-training), using videoconference supervision and online questionnaires. Six months after the end of the intervention (12 months), a follow-up assessment will also be performed to explore the potential retention of intervention effects. Since this follow-up does not directly address the main hypotheses, participation will be optional.

### Participants

A total of 122 participants with stable CVD will be included.

#### Inclusion Criteria

Participants will be adults aged 50 and older who have access to Wi-Fi connected to the internet and possess a tablet (e.g., iPad or Android) or a computer. Individuals will be included if they have stable coronary artery disease, stable chronic heart failure, corrected valvular heart disease, or atrial fibrillation, with a low-risk profile, as determined by the physician, and with no contraindication to exercise training.

#### Exclusion Criteria

1/ Non-cardiopulmonary limitation to exercise (e.g., severe arthritis) or a severe exercise intolerance. 2/ Severe respiratory disease (e.g., COPD, severe COVID-19 related symptoms, severe asthma). 3/ Significant cognitive impairment (i.e., score of 18/23 or lower on the telephone version of Mini-Mental State Examination, MMSE) (17). 4/ Contraindications to exercise testing/training (18) (e.g., uncorrected severe aortic stenosis, severe pulmonary hypertension, severe non-revascularizable coronary disease including left main coronary stenosis, significant myocardial ischemia or arrhythmia during low-intensity exercise).

### Interventional Methods

All assessments, exercise monitoring, and cognitive training will be performed remotely. Patients will be identified and recruited by Montreal Heart Institute's (MHI) physicians. During a pre-screening phone call, individuals will be asked about their interest in taking part in the study and whether they possess the proper setup to access the online tools. After a short medical questionnaire, an appointment will be fixed for a preliminary session; they will receive the information and consent form by email prior to this session. During the preliminary session, a staff member will provide details about the consent form. Participants will then be given the opportunity to provide oral consent. They will then be asked to send a written consent by email. If an oral consent is obtained, the cognitive [the telephone version of the Mini Mental State Examination; MMSE (19)] and physical (Physical Activity Readiness Questionnaire; PARQ+) screenings will be performed with a research assistant and will later be reviewed by a neuropsychologist and a kinesiologist, respectively. Once the research team receives the written consent by email and ensures eligibility, the participant will be considered enrolled. A technology tutorial will then be scheduled to ensure that internet access and tools are ready and sufficiently mastered by participants prior to the remote assessments. After baseline assessment, participants will be randomized into one of the two arms. Participants will receive automatic standardized communications by email to transmit study information like appointment scheduling, links to self-reported online questionnaires, and training guidelines. An online platform will centralize all the follow-up information. Staff members will have distinct access to either assessment or training information according to their role, to preserve the study blinding. A non-overwriting process will ensure data integrity: all form submissions will be recorded in a journal fashion.

#### Exercise and Cognitive Intervention

Participants will receive a group-specific training guide according to their randomization. This training guide contains only recommendations about the nature, intensity, and frequency of training sessions. Participants will then attend an introductory session over the phone given by their training coach.

##### Exercise Monitoring

Participants will be encouraged to complete a home-based physical exercise using video capsules available via Facebook or Youtube, created by the Centre EPIC of the MHI's team of kinesiologists (20). Aerobic, muscular strengthening, flexibility, and/or balance exercises are proposed in these videos, and do not require any equipment. Each video lasts ~15 min and includes a 5-min warm-up, followed by a 10-min training, and finally by a 2-min cool-down period. Guidelines are provided by kinesiologists to allow participants to adapt the exercise to their physical capacities. Participants will be invited to perform exercise sessions at least 5 times a week. The exercise sessions can be performed at home using the video capsules, or with other video or web-based training programs. Participants can also engage in outdoor activities (e.g., walking or cycling). To track their adherence, the duration, intensity, and nature of the activity of each training session will be reported by the participant in a journal, that they will transmit to their training coach during their weekly follow-up call. The Borg Rating of Perceived Exertion (RPE) graduated from 0 to 10 will be used by participants to assess the intensity of their exercise sessions.

##### Cognitive Training

The combined intervention will include home-based cognitive training in addition to physical exercise as described in the previous section. Participants will be asked to complete their home-based cognitive training prior to exercise training or at least 3 h post-exercise session (21). Participants will also be asked to perform their cognitive training in a comfortable position, while limiting distraction. Validated computerized cognitive training tasks, as well as video capsules will be provided. Participants will be encouraged to complete a minimum of 3 sessions per week (15–20 min/session, maximum 1 session/day), i.e., two computerized cognitive training sessions and one video capsule session. Three tasks will be available during the computerized cognitive training sessions: 1/ dual-task training, during which participants must maintain and prepare for several response alternatives and must share attention between two concurrent tasks (i.e., Dual-task), 2/ inhibition training, during which participants must refrain from giving an automatic response (i.e., Stroop task), as well as 3/ working memory training, in which participants must maintain and update information in working memory to recall items presented earlier (i.e., N-Back task). Each cognitive task lasts approximately 15 min and comprises its own sets of visual stimuli (e.g., letters, numbers, symbols) and matching button symbols. Participants will be asked to perform the tasks as fast as possible while maintaining accuracy. The three tasks will be presented in a fixed order for all participants. Task difficulty will increase gradually over the course of the participant's cognitive training to avoid task automatization and to maintain stimulation (22). Live visual feedback will be provided during training, as well as a histogram of daily performances to encourage improvement. Once per week, participants will be asked to complete a strategy-based training, via video capsules. This training is an adapted version of the MEMO+ program, which has been validated previously (23). Participants will learn different mnemotechnics (e.g., face-name association, visual imaging), and will learn about age-related changes in memory. In addition, some video capsules are targeted to help participants develop strategies to cope with multiple aspects of their daily life, such as sleep, anxiety, and nutrition. To track cognitive training adherence, participants will be asked to complete a follow-up agenda and to indicate days and times when they completed cognitive training sessions. Training adherence for computerized cognitive training tasks will also be monitored online.

#### Remote Monitoring and Coaching

Once a week, kinesiologists will call participants to (1) motivate them to stay engaged in their training, (2) ensure the safety of the exercise training sessions, and (3) to collect exercise and cognitive training data reported by participants in their follow-up agenda during the previous week. The kinesiologists will also provide updated information about training resources available online, track participant's adherence, and advise participants according to their training characteristics and study training guidelines.

#### Measurements and Outcomes

**Figure 1:**
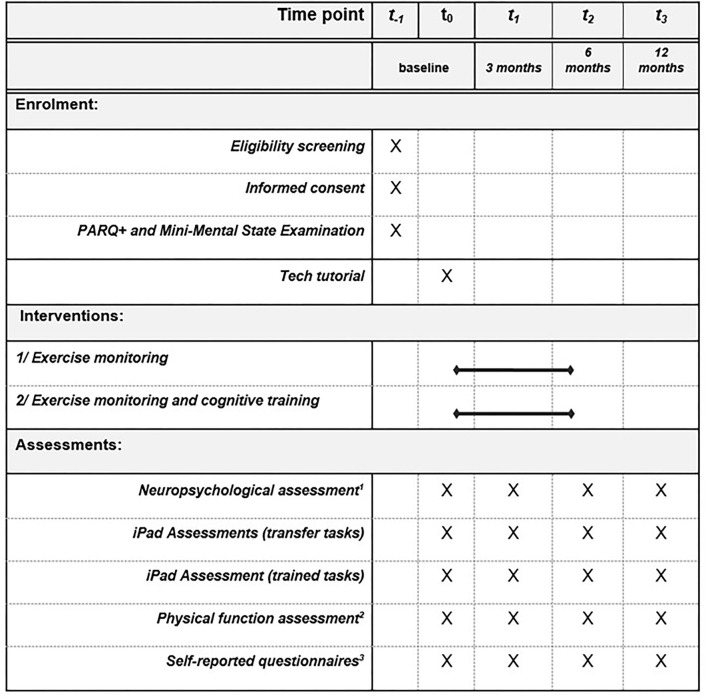
Schedule of enrolment, interventions, and assessments according to SPIRIT guidelines. ^1^Montreal Cognitive Assessment, Trail Making Test, Verbal Fluency Test, Digit Span Test, Similarity Test, Rey Auditory Verbal Learning Test. ^2^One leg balance test, 5-time Sit to Stand test, Timed up and go test, 4-meter walking speed test. ^3^Matthew questionnaire for estimated cardiorespiratory fitness, SF-12, Pittsburgh Sleep Quality Index and Berlin Questionnaire, Short Diet Questionnaire, Stait Trait Anxiety Inventory, Geriatric Depression Scale, Perceived Stress Scale, Perseverative Thinking Questionnaire, Intolerance of Uncertainty Scale, Connor Davidson Resilience Scale 10, Anxiety Sensitivity Index, Social and Community Involvement Questionnaire, Lubben Social Network Scale. The following questionnaires will be completed at baseline only: Medical questionnaire, Q-COVID questionnaire, Physical Activity Scale for the Elderly, Cognitive Reserve Questionnaire, Bem sex-role Inventory.

Participants will complete videoconference assessments prior to the intervention, mid-intervention (3 months), post-intervention (6 months), as well as 6 months following the end of the intervention (12-month follow-up). All the testing procedures are reported in Figure 1. At baseline, demographics, and medical information, including chronic diseases, comorbidities, and medications will be collected by medical questionnaire. A COVID-19 questionnaire (i.e., QCOVID) will address the social and financial consequences of the COVID-19 pandemic on participants, as well as its impact on their physical activity routines.

##### Primary Outcome: Cognitive Performances

The primary outcome of the study will be cognitive performances. Participants will be tested for general cognitive functioning using a remote version of the Montreal Cognitive Assessment (MoCA) (24). Also, three main components of cognition will be assessed with a comprehensive neuropsychological evaluation, from which composite z-scores will be computed: executive functions, processing speed and episodic memory. The neuropsychological assessment will include the following tests that will be administered in a predetermined order: Rey auditory verbal learning test, Digit Span, oral Trail Making Test (25), Phonological and Semantic Fluency from the D-KEFS battery (26), and Similarities subtest from the WAIS-IV (27). The neuropsychological assessment, including the MoCA, will be performed via videoconference (Zoom). All tests have been validated for remote administration and have previously been used in large participant cohorts (28). Importantly, these tests are validated and normed for an older adult population (17, 21, 29). The similarities subtest will only be completed at baseline to obtain a measure of crystallized intelligence; its completion will be optional and performed according to participant's fatigue. Participants will also realize a computerized or tablet-based cognitive assessment (30). The three computerized cognitive tasks include a dual-task (30), Stroop task, and N-back. Participants will complete trained and transfer versions (same tasks but with different stimuli) that will be presented in a counterbalanced order between participants, via a latin-square procedure. Comparing trained and transfer tasks allows to quantify participants' ability to transfer cognitive training effects to novel tasks. Subcomponents of each task allow dissociating different attentional control mechanisms from mere cognitive speed. Importantly, the tasks record response time in milliseconds, which reduces the likelihood of ceiling effects. These tasks also tend to be more sensitive to training effects. Finally, computer equipment (i.e., computer or tablet) used by participants for the computerized tasks will be documented. Participants will be asked to use the same computer equipment for the four testing periods (including the 12-month follow up).

##### Secondary Outcome: Physical Functions

Physical functions will be assessed remotely by videoconference. First, balance will be assessed with a one-leg balance test (i.e., stay on one leg as long as possible for a maximum of 60 s; recorded twice for both legs). Lower limb muscle strength will be evaluated with the Five-Time-Sit-to-Stand test (i.e., five consecutive sit-to-stand movements performed as fast as possible without using arms as leverage). The 4-meter walk test and the Timed Up-and-Go (TUG) performed at spontaneous and fast speeds will be used to assess mobility. During the 4-meter walking test, participants' walking speed will be measured in a straight line over 4 meters. During the TUG, participants will be timed for the following sequence: getting up from a chair, walking 3 meters, turning around, and sitting back in the chair. These four tests are commonly used in a clinical setting and have demonstrated their associations with global cognition and specific cognitive domains such as processing speed and executive functions (31). During the physical function session, participants will be first questioned about their history of musculoskeletal and sensory conditions to ensure that they can safely execute the tests. Prior to the physical function assessment, participants will receive by email with a detailed description of each test with its set-up and demonstration videos. Then, at the beginning of the session, the testing set-up will be verified by the research assistant or kinesiologist, to ensure there is sufficient space to perform the tests, as well as a sufficient accuracy for both 4 and 3-m of the walking and TUG tracks measures. During the testing, adequate visual feedback will always be maintained to ensure participant's security and reliability of the measures. Finally, to complete the physical function assessment, the Matthews questionnaire will be used to predict participants' cardiorespiratory fitness (i.e., VO2_max_), which is based on sex, age, anthropometric data, and self-reported physical activity level (32).

##### Tertiary Outcomes

The following tertiary outcomes will be measured with validated self-reported questionnaires that participants will fill in through online forms: **1/ Sleep:** Participants will complete the Pittsburgh Sleep Quality Index (PSQI) and the Berlin Questionnaire. PSQI scores range from 0 to 21, a lower score indicating better sleep quality. The Berlin Questionnaire will determine the risk of sleep apnea. **2/ Anxiety/mood:** The Geriatric Depression Scale (GDS), the Perceived Stress Scale, the SF-12 (quality of life), the State-Trait Anxiety Inventory, the Anxiety Sensitivity Index, the Intolerance of Uncertainty scale, the Perceived vulnerability to disease, the Connor-Davidson Resilience Scale 10, and the Perseverative Thinking Questionnaire, will be used to assess mood and anxiety. **3/ Cognitive reserve:** will be evaluated at baseline. To do so, participants will provide their total years of education and will complete a modified version of the Rami and colleague's cognitive reserve questionnaire (33), adapted for French and English by the CIMA-Q team (17, 34). **4/ Social support:** Social and Community Involvement Questionnaire (35) and the Lubben Social Network Scale will be completed. **5/ Diet:** Participants will complete the Short Diet Questionnaire (36). **6/ Prior physical activity:** Participants will fill in the Physical Activity Scale for the Elderly (37). **7/ COVID-19 impact:** The QCOVID questionnaire will be completed at baseline only. **8/ Gender:** The Bem Sex-Role Inventory will be filled at baseline only. All questionnaires will be filled in by participants using anonymous online forms.

##### Research Plan

Following the preliminary call, five videoconference sessions will be scheduled with the participants. The first session will be a technology tutorial that will ensure that internet connectivity and tools are ready and mastered enough by participants to engage in testing. Then, participants will complete 4 pre-testing sessions (T0), 4 mid-testing sessions (T1 at 3 months), 4 post-testing sessions (T2 at 6 months), and 4 optional follow-up sessions (T3 at 12 months). Total participation in this study is expected to be of a 12-month duration, including 6 months of intervention (Figure 1).

### Data Analysis

#### Sample Size Calculation

The team of biostatisticians from the Montreal Health Innovations Coordinating Centre (MHICC) performed the sample size calculation on the primary outcome, i.e., the differential effect of physical exercise training alone vs. the combined physical and cognitive training on cognitive performance (executive functions). The calculation was based on existing values available in the literature for physical exercise training alone, as well as on different plausible effect sizes for the combined physical and cognitive training (no existing values in the literature for this group), and on clinically relevant effect sizes. The calculation revealed that a sample size of 49 subjects in each group will have 80% power to detect a difference in means of−0.186 (difference between physical training alone mean of 0.239 and combined training mean of 0.425, for an effect size of 0.624), assuming that the common standard deviation is 0.325 (slightly larger than in a traditional context because of the home-based administration of neuropsychological tests) using a two-group *t*-test (0.050 two-sided significance level). As we expect a 20% attrition rate based on our previous exercise studies, we will thus recruit 61 participants per intervention arm, for a total of 122 patients with CVD. Participants will be randomized in one of the 2 arms: home-based physical training alone or combined physical and cognitive home-based training. A biostatistician from the MHICC generated the randomization sequence.

#### Statistical Analysis

The variables in the study will be presented using descriptive statistics. The mean, standard deviation, median, minimum, Q1, Q3 and maximum will be presented for continuous variables. The number and percentage will be presented for nominal/ordinal variables. The assumptions of the statistical tests will be examined, and data transformation or non-parametric analyses may be used as appropriate. SPSS and SAS software version 9.4 or higher will be used to conduct the analyses.

Mean changes in cognitive performance from baseline will be analyzed using a repeated measures analysis of covariance (ANCOVA) model, adjusted for age, sex, education, including the effects of the intervention as between-subject variable (physical exercise alone or combined with cognitive training) and time as within-subject variable (pre at baseline, mid at 3 months, post-intervention at 6 months). Observation of a statistically significant difference in the primary outcome between pre- and post-intervention timepoints will be considered as the evidence of the intervention's efficacy (i.e., primary hypothesis). The interaction between intervention arms (i.e., physical exercise alone or combined training) and performance changes from pre to post-intervention will address the added value of cognitive training compared to physical exercise alone (i.e., secondary hypothesis). Secondary and exploratory outcomes will be analyzed as the primary outcome.

#### Blinding

Assessors performing the evaluations and investigators will be blinded to group allocation. The statistician will be blinded until completion of the statistical analyses. Only the kinesiologists executing the weekly follow up of exercise/cognitive training program will be aware of the assigned intervention. Kinesiologists will not take part in any assessment.

## Discussion

The latest WHO Guidelines on physical activity and sedentary behavior recommends for adults and older adults with chronic conditions, such as CVD, at least 150–300 min of moderate-intensity aerobic physical activity, or at least 75–150 min of vigorous intensity aerobic physical activity, or an equivalent combination of moderate- and vigorous-intensity activity throughout the week (30). With the COVID-19 crisis and the confinement measures in place (e.g., closed gyms), these recommendations are difficult to meet (1, 38, 39). Among the 1,098 Canadian adults included in the study by Lesser et al., 40% of inactive individuals became less active during the restrictions of daily living caused by the COVID-19 pandemic (39). In cardiac patients, who already tend to have a sedentary/inactive profile, the confinement measures excessively increase the level of physical inactivity and sedentary lifestyle, which can further deteriorate cardiovascular health and increase the risk of acute events (4). Frail individuals, older adults, and individuals with chronic conditions such as CVD are at higher risk to suffer from the direct and collateral consequences of the COVID-19 pandemic. Hence, allowing older adults with CVD to maintain and/or improve their cognitive and physical health may help to prevent a possible secondary crisis caused by the long-term alteration of their health condition. Our home prevention strategies allow patients, including those living in remote areas, to have cognitive and physical stimulation for 6 months.

Exercise and cognitive training separately have demonstrated enhancements of multiple and specific aspects of cognitive and physical functions and could have a synergistic effect on cognition in healthy older adults (15). Individuals with CVD who participated in a CR program improve their cognitive performances (12). In addition, the synergistic effect of exercise associated with cognitive training on cognition has never been shown in older adults with CVD. Thus, some questions need to be addressed regarding how much benefits can be gained by adding cognitive training to physical exercise and regarding the optimal dose of each type of intervention.

This trial proposes crisis-adapted lifestyle interventions and remote assessments. More precisely, to further ensure the continuity and the regularity of the exercise training, the proposed trial offers weekly phone call support and an online video training program, providing a wide range of exercises to help participants maintain their exercise routine even during the lockdown. In CVD, among the patients who complete a phase II center-based CR about half of the patients tend to return to their previous lifestyle habits, become sedentary again after a few months, and are non-adherent to physical activity target levels recommended by physicians (40, 41). Home environment with regular contact with telephone support or videoconference from the medical or research team may be more likely to help sustain positive physical and psychosocial changes over time, compared to institution-based programs (42–44).

Most participants in exercise clinical trials come from urban areas, which likely limits generalization of findings, and individuals from rural communities are still underrepresented (45). Home-based interventions, made available for all, would address this problem, and offer a great solution to counteract sedentary/inactivity behavior in the COVID-19 pandemic situation. A home-based combined intervention with exercise and cognitive training could help to maintain and enhance cognition and cardiovascular health in older adults with CVD who could no longer access training facilities during the COVID-19 lockdown.

### Study Limitations

Some limitations of our trial need to be mentioned. First, our design does not include a control group without intervention. Because of the pandemic context, some ethical considerations refrained the research team from implementing a control group without any intervention. However, to partially address this limitation, in our analysis we will take into account the dose response effect as a function of the volume of training performed by participants. Based on the nature of each activity, its intensity and its frequency, a weekly dose of physical exercise (converted in kcal and in METs using the Compendium of Physical Activities) will be computed. The effect of physical exercise dose on cognitive and physical functions will be evaluated according to participant's mean weekly dose during the 6 months of intervention. Based on the overall study sample distribution, participants could be classified into “higher,” “medium,” and “lower” doses of physical exercise (i.e., tercile split).

Secondly, our participants do not have precise control of the intensity and duration of the exercise sessions (heart rate monitors, pedometers, or other tools). This is only declarative data reported weekly with the kinesiologists. Our study focuses on exercise and cognitive training in CR's phase III and should not be confused with a comprehensive CR program delivered in phase II. Adherence to medication, diet and appropriate teaching about patient's risk factors and CVD are critical aspects of a comprehensive CR program (phase II). Consequently, we will include some patients who have completed a CR program and others who have not benefited from this program. Then, non-exercise prediction of cardiorespiratory fitness with an equation is valid for healthy individuals, but little is known about its reliability in patients with CVD. Moreover, variability was observed in the accuracy between non-exercise prediction equations and the ability of equations to detect changes in cardiorespiratory fitness (46). Therefore, the use of non-exercise prediction equations could lead to a significant error in the estimated change of participants' cardiorespiratory fitness. Finally, sex was not taken into account in recruitment and randomization processes, although sex differences have been reported on cognition (47), as well as in exercise-training effects on cognition in healthy older adults (48). No study to date has documented potential sex-related differences with regards to the effects of exercise, cognitive or combined training on cognition and physical outcomes in older adults with CVD. Given the sex-related differences in cognition, exercise intervention effects and diseases, sex differences are to be expected in the present study.

### Trial Status

The COVEPICARDIO study is the fourth version of the protocol validated by the Research Ethics Board of Montreal Heart Institute in October 2020. Recruitment for the study started May 20, 2020 and is planned to be finished in September 2021.

## Ethics Statement

The study protocol was approved by the Montreal Heart Institute's (MHI) Research Ethics Board (FWA00003235; research project: ICM 2020-2785). All participants will provide informed consent prior to starting the study.

## Author Contributions

LB, FB, ED, CG, MG, TV, C-AB, C-AG, KS, and PV: conception and design of the research. LB: principal investigator. AN: co-investigators. FB, ED, CG, MG, TV, C-AB, C-AG, KS, BB, MO, and PV: collaborators. FB wrote the first version of the manuscript, drafting, and revision of the manuscript. All authors revised it and contributed significantly to write the final version that was accepted.

## Funding

This study is supported by the Montreal Heart Institute Foundation and the Mirella and Lino Saputo Research Chair in Cardiovascular Health and the Prevention of Cognitive Deficits from University of Montreal at the Montreal Heart Institute and the Montreal Heart Institute Foundation.

## Conflict of Interest

The authors declare that the research was conducted in the absence of any commercial or financial relationships that could be construed as a potential conflict of interest.

## Publisher's Note

All claims expressed in this article are solely those of the authors and do not necessarily represent those of their affiliated organizations, or those of the publisher, the editors and the reviewers. Any product that may be evaluated in this article, or claim that may be made by its manufacturer, is not guaranteed or endorsed by the publisher.
